# Estrogen Regulation of MicroRNA Expression

**DOI:** 10.2174/138920209788185289

**Published:** 2009-05

**Authors:** Carolyn M Klinge

**Affiliations:** Department of Biochemistry & Molecular Biology, Center for Genetics and Molecular Medicine, University of Louisville School of Medicine, Louisville, KY 40292, USA

**Keywords:** MicroRNA, miRNA, estrogen, estrogen receptor.

## Abstract

Women outlive men, but life expectancy is not influenced by hormone replacement (estrogen + progestin) therapy. Estrogens appear to protect brain, cardiovascular tissues, and bone from aging. Estrogens regulate genes directly through binding to estrogen receptors alpha and beta (ERα and ERβ) that are ligand-activated transcription factors and indirectly by activating plasma membrane-associated ER which, in turns, activates intracellular signaling cascades leading to altered gene expression. MicroRNAs (miRNAs) are short (19-25 nucleotides), naturally-occurring, non-coding RNA molecules that base-pair with the 3’ untranslated region of target mRNAs. This interaction either blocks translation of the mRNA or targets the mRNA transcript to be degraded. The human genome contains ~ 700-1,200 miRNAs. Aberrant patterns of miRNA expression are implicated in human diseases including breast cancer. Recent studies have identified miRNAs regulated by estrogens in human breast cancer cells, human endometrial stromal and myometrial smooth muscle cells, rat mammary gland, and mouse uterus. The decline of estradiol levels in postmenopausal women has been implicated in various age-associated disorders. The role of estrogen-regulated miRNA expression, the target genes of these miRNAs, and the role of miRNAs in aging has yet to be explored.

## INTRODUCTION

Women live longer than men. Estrogens (estradiol, estrone, and estriol) are steroid hormones that regulate development and homeostasis in a wide variety of tissues including the brain, reproductive tract, vasculature, and breast. Estradiol (E_2_) is synthesized in the ovary and is the primary estrogen in premenopausal women. Animal studies have shown that higher estrogen levels in females protect against aging by upregulating the expression of antioxidant, longevity-related genes, *e.g*., selenium-dependent glutathione peroxidase (GPx) and Mn-superoxide dismutase (Mn-SOD) [1], by protecting against stroke-related injury [2], by vasorelaxing effects [3], by direct myocardial protection [4], and by activating the insulin receptor substrate (IRS)-1 signaling pathway [5]. Women’s life expectancy seems not to be influenced by hormone replacement therapy (HRT = estrogens plus a progestin, usually conjugated equine estrogens and medoxyprogesterone acetate (MPA)) in postmenopausal women, but atherosclerosis and bone loss are considerably delayed. In addition, HRT protects against Alzheimer’s disease (AD) [6], perhaps by suppressing elevated gonadotropin levels, *i.e*., luteinizing hormone (LH), in postmenopausal women since elevated LH is thought to play a key role in AD pathogenesis [7]. Other age-associated impairments are also reduced by estrogen. For example, premenopausal women have a reduced risk for cataracts compared with men of the same age group and women in the Farmingham study who used estrogen replacement therapy (ERT) showed reduced risk for cataracts [8]. miRNAs are a class of naturally-occurring, small, non-coding RNA molecules that are related to, but distinct from, small interfering RNAs (siRNAs) which regulate mRNA translation or stability [9-11]. There are very few studies on the hormonal regulation of miRNAs expression. Select changes in microRNA (miRNA) expression correlate with diagnostic markers used in breast cancer therapies, *e.g*., estrogen receptor α (ERα) and tumor grade [12-22]. However, there are only 5 reports that E_2_ regulates miRNA expression that will be reviewed here. It is highly likely that hormones play a major role in regulating miRNAs by both genomic (transcriptional) and non-genomic mechanisms of action. Identification and characterization of estrogen-regulated miRNAs may provide new biomarkers and therapeutic targets in aging as well as in diseases including breast cancer.

### Genomic ER Activities

Initiation of transcription is a complex event occurring through the cooperative interaction of multiple factors at the target gene promoter. I will use the term ER to refer to either ERα or ERβ or to both subtypes. I will refer to each subtype individually as pertinent to the known functions of these two proteins. Estrogen action is primarily mediated through binding to ER. ERα and ERβ are members of the steroid/nuclear receptor superfamily of proteins of which there are 48 members in mammals [23]. ERα and ERβ are highly conserved within the DNA binding domain (DBD, C domain), but differ in their N- and C- termini [24]. Structurally, ER has 6 domains lettered A-F from N- to C-terminus. ER is believed to be the ancestral steroid receptor originating 600-1200 million years ago, presumably because of the role of estrogens in reproduction and maturation [25, 26].

In the simplest model, the binding of estradiol (E_2_) *via *hydrogen bonding to residues within the ligand binding pocket of the ligand binding domain (LBD, E domain) results in conformational changes termed activation [27]. These conformational changes induced by E_2_ binding result in loosening of contact between the N-terminus and the C-terminus of ERα and exposes nuclear localization signals within the DNA binding domain (DBD, C domain) and hinge region (D domain) as well as altering structure of the LBD [28]. Crystal structure studies of the LBD of ERα excluding the F domain shows that the LBD has 12 alpha helices and E_2_ binding repositions helix 12 such that activation function-2 (AF-2) is exposed [29]. Helix 12 acts as a “switch” controlling accessibility of the coregulator interaction site. Ligand binding also facilitates ER dimerization (homodimerization or heterodimerization of ERα and ERβ).

E_2_-liganded ER interacts directly with a specific DNA sequence called the estrogen response element (ERE = 5’-AGGTCAnnnTGACCT-3’), historically located in the promoter region and currently established to be located at great distances from the transcription start site including in the 3’ flanking regions of target genes [30-34]. DNA binding increases ER interaction with basal transcription factors and coregulator proteins (reviewed in [35]). Fig. (**1**) depicts essential features of genomic ER action. EREs are enriched in genes upregulated by E_2_-ERα, at least in MCF-7 cells [36]. ERα can also be activated by phosphorylation of ser118 in the N-terminal A/B domain that activates activation function-1 (AF-1) in the absence of ligand binding [37]. At least in HeLa cells transfected with fluorescent fluorescent-tagged ERα (GFP- or CFP- ERα), E_2_ causes rapid intracellular and intranuclear movement of ERα to form punctuate nuclear speckles that appear to indicate ERα-nuclear matrix interaction [38-40]. In addition to direct ER-ERE binding, ER also activates transcription *via *a “tethering mechanism” in which ER interacts directly with transcription factors, *e.g*. Sp1 [41] and AP-1 [42], bound to their response elements. These DNA-protein and protein-protein interactions recruit coactivator/chromatin remodeling complexes resulting in histone modifications that lead to nucleosomal remodeling, increased accessibility to the DNA template for RNA polymerase II interaction, and increased target gene transcription (reviewed in [43-45]).

By definition, coactivators are proteins that interact directly with transcription factors to enhance transcription [46]. It is important to note that the term coactivator or corepressor is used when referring to an ER (or other NR) coregulator is gene-, cell-type, and context- specific [47]. This indicates that proteins classified as coactivators can also repress transcription and corepressors such as SMRT are gene- and cell- specific coactivators for ER [48]. Coactivators promote the assembly of the transcription initiation complex in part by altering chromatin structure and ‘loosening’ DNA-histone interactions, facilitated by increased histone lysine residue acetylation, methylation, ubiquitination, or sumoylation [49]. Once the transcription initiation complex is complete, RNA polymerase II (RNA pol II) is recruited to the transcription start site and begins transcription. By my count, at least 60 different ER coactivators and 23 corepressors have been functionally identified (reviewed in [43, 50, 51], see also http://www.nursa.org/index.cfm). The current model predicts that ERE-bound, agonist-liganded ER recruits coactivator proteins to enhance gene expression [52]. In contrast for those genes at which tamoxifen (TAM) is an antagonist of ER transactivation, the LBD of TAM- occupied ERα does not interact with coactivators due to key conformational differences between agonist and antagonist – occupied ERα in AF-2 [53]. TAM-occupied ERα interacts with corepressors, *e.g*. NCoR or SMRT [54-58], that recruit histone deacetylase (HDAC) complexes thus keeping chromatin condensed and blocking transcriptional activation. Antiestrogen ICI 182,780 (Fulvestrant)-occupied ERα is targeted to the 26S proteasome for degradation [40, 59]. In contrast, although ICI 182,780 inhibits ERβ-mediated transcription, it stabilizes the ERβ protein [60].

### Nongenomic Estrogen Action

In addition to its classical genomic/transcriptional effects mediated by ER-DNA interaction, described above, E_2_ has rapid “nongenomic, extra-nuclear, or membrane-initiated” effects that occur very rapidly, *i.e.*, within seconds-minutes after E_2_ administration [61-64]. These effects are independent and distinct from the genomic*, i.e*., ER-mediated transcription, activities reviewed in the preceding section. Fig. (**1**) highlights some of the established nongenomic activities of ER. Rapid estrogen-stimulated intracellular activities are mediated by plasma membrane (PM)-associated ER and/or by an ‘orphan’ G-protein coupled receptor GPR30 [65]. Evidence of a PM-associated ER population is supported by experiments in which a cell-impermeable E_2_–bovine serum albumin or other E_2_-conjugate was shown to rapidly initiate intracellular kinase cascade activities including MAPK/ERK (p42/p44 MAPKs), endothelial nitric oxide synthase (eNOS), and PI3K/AKT [66-75]. In MCF-7 cells, E_2_ rapidly increased PIP2-phospholipase C activity [76], mobilized intracellular Ca^2+^, activated MAPK [62, 77-96], and PI3K/AKT [87, 97-102]. Immunohistochemical techniques have visualized membrane ERα in a variety of cell types including endometrial and breast cancer cells [103], pituitary cells [104], and lung cancer cells/tumors [91]. ERβ was shown to be located in the PM of primary cortical neurons in recent confocal imaging studies [105]. Overall, the PM localization of ER appears to be cell type-specific.

Premenopausal women have a lower risk of developing cardiovascular disease [106, 107] and hypertension than men or post-menopausal women and estrogens are thought to be responsible for regulating peripheral resistance [108] as well as effects in the myocardium [106]. Many of the cardioprotective activities of E_2_ may be mediated by nongenomic signaling. Cumulative studies show that a subpopulation of intact ERα is associated with the endothelial PM and with caveolae [61]. Recent electron microscopy studies revealed nuclear, cytoplasmic, and plasma membrane localization of both ERα and ERβ in human umbilical vein endothelial cells (HUVEC) [109]. ERα [79, 110] has been shown to interact with caveolin-1 (Cav-1) which serves as a structural core for interaction of plasma-membrane-associated proteins including the α-subunit of G-proteins, Ha-Ras, Src-kinases, eNOS, epidermal growth factor (EGF) receptors, and some protein kinase-C isoenzymes [110]. E_2_-ERα interaction within caveolae leads to Gαi activation, MAPK and Akt signaling, and perturbation of the local Ca^+2^ environment, leading to eNOS phosphorylation and NO production [61]. Endothelial PM-associated ERα is coupled *via *a Gαi to MAPK and eNOS [111].

The mechanism by which ERα and its splice variant ERα46 localizes to the PM involves palmitoylation [112-115]. In addition, ER interaction with intracellular proteins is important in PM association. In MCF-7 cells, the adaptor proteins Shc shuttles ERα from the nucleus to the PM where ERα interacts with the IGF-1 receptor (IGF-1R) [89, 96]. Similarly, another scaffold protein, called MNAR (modulator of nongenomic activity of ER), has been implicated in ERα-cSrc interaction and MAPK signaling [85, 95, 98, 116-128]. A role for a membrane caveolae-localized ERα46 in rapid NO release *via *PI3K/Akt activated eNOS has been reported in endothelial cells [112, 123, 129, 130].

In addition to PM-associated ER, GPR30 has reported to serve as a membrane estrogen receptor because it binds E_2 _with high affinity (Kd = 2.7nM) and activates adenylate cyclase, thus increasing cAMP levels [70, 73, 131-134]. GPR30 is distinct from ERα and ERβ in that ICI 182,780 and tamoxifen also bind GPR30 with high affinity and mimic the effects of E_2_ [133]. Although the role of GPR30 in MCF-7 and SKBr3 breast cancer cells has been questioned [62], it appears likely that GPR30 is a *bone fide* membrane estrogen receptor in some cell types [135-141].

### MicroRNAs (miRNAs)

Evidence from the Encyclopedia of DNA project (ENCODE) has revealed surprising new information about the human genome. For example, although the protein coding regions account for only 2% of the total DNA in the human genome, surprisingly, 80-93 % of the genome is “expressed” [142-144]. The transcribed RNAs are largely conserved between humans and mice suggesting that these noncoding RNAs (ncRNAs) have important functions. Evidence of the importance of the various types of ncRNAs was recently reviewed [145] and includes roles in cancer, diabetes, and coronary disease, all aging-associated disorders. Among the small ncRNAs are microRNAs (miRNAs). The importance of miRNAs is highlighted by the fact that the 2008 Albert Lasker Award for Basic Medical research was awarded to Drs. Victor Ambros [146] and Gary Ruvkun [147] who discovered and characterized the first miRNAs in *C. elgans* and Dr. David Baulcombe who discovered let-7 miRNA in plants [148] (see also http://www.laskerfoundation.org/).

miRNAs are a class of naturally-occurring, small, non-coding RNA molecules that are related to, but distinct from, small interfering RNAs (siRNAs) [9-11]. About half of miRNAs are expressed from introns of protein-coding transcripts and miRNAs have 5' and 3' sequence features that form boundaries including transcription start sites, CpG islands, and transcription factor binding recognition elements [149]. miRNAs may be differentially processed from the sense and antisense strands of the same hairpin RNA or transcripts from the same locus, thus expanding the number of miRNAs from a single genomic locus [145].

The pathway of mature miRNA biogenesis is depicted in Fig. (**2**). miRNA genes are mostly transcribed by RNA polymerase II into primary-micro-RNAs (pri-miRNAs) that are capped and polyadenylated [150]. Pri-microRNAs contain self-base-pairing stem-loop structure that is necessary for critical processing within the nucleus by Drosha, an endonuclease of the RNAse III family, and its cofactor DGCR8 into short (60 to 70 nt) imperfect hairpin structure precursor-miRNAs (pre-miRNAs) [151]. These pre-miRNAs are exported from the nucleus by exportin and Ran-GTP. Pre-miRNAs are processed by the cytoplasmic RNAse II enzyme Dicer to form mature ~22 nt transiently double-stranded miRNA duplexes that are transferred to Argonaute proteins (Ago1, Ago2, Ago3, and Ago4 [152]) in the RNA-induced silencing complex (RISC), leading to unwinding of the duplexes to form single stranded microRNAs. RISC guides RNA silencing with the miRNA binding either to the 3’ untranslated region (3’ UTR) or to the open reading frame (ORF) of its target mRNA [153-156]. Most commonly, because of imperfect complimentarity of the base pairing between the miRNA and the 3’UTR, the RISC complex causes translational repression by RISC interaction with eIF6 which prevents assembly of 80S ribosomal assembly [157] or by inhibition of translation [16]. Thus, miRNA-mRNA 3’UTR interaction results in a decrease in target protein, not mRNA. The 7 to 8 nucleotide region of basepairing between the 5’ end of the mature miRNA and the mRNA is called the ‘seed sequence’. Base pairing of the miRNA-RISC complex within the ORF requires almost perfect complimentarity for its mRNA target and the mRNA is either degraded or translation is blocked [150]. The miRNA-containing ribonucleoprotein particle (miRNP)-silenced mRNA is directed to the P-bodies, where the mRNA is either released from its inhibition upon a cellular signal and/or actively degraded [158]. Comparative genomics analyses have revealed that over 45,000 miRNA binding sites within human 3'UTRs that are conserved above background levels [159]. This number was reported to indicate that more than 60% of human protein-coding genes have been under selective pressure to maintain pairing to miRNAs [159]. Recent evidence indicates that miRNAs may also increase translation of select mRNAs in a cell cycle-dependent manner [160].

miRNAs have been demonstrated to play important roles in regulating various cellular processes including replication, differentiation, and apoptosis [11, 155, 161-176]. Since each of these processes play a role in aging, it is reasonable to suggest that altered expression and function of miRNAs regulate aging. The role of miRNAs in aging is completely unexplored. The human genome contains > 700-1,200 miRNAs http://microrna.sanger.ac.uk/sequences/ [149] and miRNAs are expressed in a tissue-specific manner [177]. Each miRNA targets ~ 200 transcripts directly or indirectly [178], but the *bone fide* physiological targets of the vast majority of miRNAs is virtually unknown.

### Altered miRNA Expression in Breast Cancer

The spectrum of miRNAs expressed in solid tumors, *i.e*., prostate, colon, stomach, pancreas, lung, and breast, is different from normal tissues [177]. Although the precise sequence of events leading to breast tumors is not understood, lifetime exposure to estrogens is widely accepted as a major risk factor for the development of breast cancer [179]. Some investigators have documented that E_2_ is carcinogenic in human breast epithelial cells [180-182]. However, epidemiological evidence disputing the carcinogenicity of E_2_ in humans has been published [183]. Surprisingly, there are no published studies evaluating the effect of E_2_ on global miRNA expression in breast cancer cells.

Aberrant patterns of miRNA expression have been reported in human breast cancer [12, 13, 18, 20, 21, 151, 162, 164-170, 176-178, 184-191] and recently reviewed [150]. The first miRNA study in breast cancer indicated differential expression of miRNAs in concordance with other well-established markers of breast cancer stage and patient prognosis including ERα and PR expression, tumor stage, number of positive lymph nodes, and vascular invasion [20]. Different miRNA expression profiles were also associated with ErbB2+ *versus* ER+ tumors [22]. More recently, patients whose breast tumors showed reduced miR-126, miR-206, or miR-335 were found to have reduced survival, regardless of ERα or ErbB2 status [18].

A number of genes involved in breast cancer progression have been identified by *in silico* analysis to be targets of miRNAs that are deregulated in breast cancer [192] and some have been experimentally proven. A recent study reported that miR-21 expression was reduced in breast tumors and that antisense to miR-21 suppressed MCF-7 breast cancer cell growth *in vitro* and as tumor xenografts in mice by regulating Bcl-2 [13]. Interestingly, we recently reported that overexpression of miR-21 in MCF-7 cells increased soft agar colony formation, reflecting increased tumorigenicity of these cells [193]. We demonstrated that miR-21 binds to a seed element in the 3'-UTR of the programmed cell death 4 (PDCD4) gene and reduces Pdcd4 protein expression [193].

The breast cancer oncogene/coactivator AIB1/SRC-3/NCOA3 is regulated by mir-17-5p and there is a reciprocal relationship between reduced miR-17-5p and increased AIB1 in breast cancer cells [194]. Overexpression of miR-17-5p reduced E_2_-stimulated proliferation of MCF-7 breast cancer cells, indicating a role for deregulation of miR-17-5p in breast cancer [194]. Overexpression of miR-125a and miR-125b decreased ERBB2 and ERBB3 mRNA and protein levels, inhibited phosphorylation of ERK1/2 and AKT, and inhibited the anchorage-independent growth of ERα-negative/ErbB2-overexpressing SKBR3 breast cancer cells [195]. ERα mRNA stability is negatively regulated by miR-206 in MCF-7 cells and miR-206 expression is higher in ERα negative MDA-MB-231 cells [162].

### Estrogenic Regulation of miRNA Expression

A PubMed search for estrogen AND miRNA revealed 27 papers. However, in that list and in total there are, to my knowledge, only 6 studies in which miRNA regulation by E_2_ has been directly examined (see below). Indeed, although a software application that will retrieve all miRNA:mRNA functional pairs in an experimentally derived set of genes was recently developed and used to identify E_2_-regulated mRNA genes in breast cancer [196], this paper does not experimentally address miRNA changes regulated by E_2_.

### E_2_ Regulation of miRNAs in Animal Studies

The effect of E_2_ in miRNA expression has been examined in zebrafish [197], August Copenhagen Irish (ACI) rats [198], and mouse splenocytes [199]. A recent review of miRNA expression in female mammalian reproductive tissues described transgenic and knockout mouse models and findings related to changes in miRNAs in the ovary and uterus in response to deletion of Dicer [200], LH, and during development (immature versus mature mice) [201]. Changes in miRNA expression in mouse uterus during implantation have been cataloged [202]. Importantly, the authors of this review concluded that the expression, regulation, and function of miRNAs within specific tissues and cells still needs to be determined [201].

A study of the effect of E_2_ on miRNA expression in the adult (3 mos) zebrafish male (*Danio rerio*) identified altered expression of 38 miRNAs in the whole body homogenates [197]. E_2_ was added to the aquariums at a final concentration of 5 µg/liter (18 nM) and although various times of treatment were analyzed, most miRNA changes in response to E_2_ were observed after 12 h. miRNAs were regulated by E_2_ in a tissue-specific manner with E_2_ downregulating miRNAs in the liver and increasing miRNA expression in the skin of the zebrafish. For example, miR-122 was decreased by E_2_ in skin, but increased in gills, intestine. and liver. Among the most up-regulated miRNAs were miR-196b and let-7h, and miR-130c and miR-101a were the most down-regulated. The authors identified *Hoxb8a* as a target of miR-196b and showed that E_2_, by increasing miR-196b, decreased Hoxb8a [197]. The authors concluded that miR-196b may serve as “a biomarker of exposure to environmental estrogens and endocrine-disrupting chemicals that fish may encounter in their aquatic environment” [197].

In another study, miRNA expression was analyzed after 6, 12, and 18 weeks of E_2_-induced mammary carcinogenesis in female ACI rats [198]. After 6 and 12 wks of E_2_ exposure, 15 miRNAs were down-regulated, *e.g*., miR-22, miR-99a, miR-106a, miR-127, miR-499, and 19 miRNAs were-up-regulated, *e.g., *miR-17-5p, miR-20a, miR-21, miR-129-3p, miR-106a, miR-22, and miR-127. By 18 wks of E_2_ treatment, the mammary glands were characterized by lobular involution and hyperplasia, and only 1 miRNA was down-regulated (miR-139) and 5 miRNAs were up-regulated (miR-20b, miR-21, miR-103, mir-107, miR-129-3p, and miR-148a). Genes targeted by three of the altered miRNAs were examined: miR-20a regulates E2F1, miR-106a regulates RBI, and miR-127 regulates BCL6. Western blot of mammary gland lysates after 12 wks of E_2_ showed that levels of RBI and E2F1 were decreased and BCL6 protein was increased, data that are in agreement with the increase miR-20a and miR-106a and the decrease in miR-127 detected [198].

E_2_ decreased miR-146a, miR 125a, miR-125b, let-7e, miR-126, miR-145, and miR-143 and increased miR-223, miR-451, miR-486, miR-148a, miR-18a, and miR-708 expression in mouse splenic lymphocytes [199]. Notably, transfection of cells with miR-146a decreased LPS-induced IFNγ. AS to miR-223 blocked LPS-induced IFNγ secretion in splenocytes from E_2_ treated mice. This is the first report on E_2_ regulation of miRNA expression in immune cells [199], but provided no mechanism by which E_2_ regulated these changes.

### E_2_ Regulation of miRNAs in Human Cell Lines

E_2_ and the ERα-selective agonist 4,4',4''-(4-Propyl-[1H]-pyrazole-1,3,5-triyl)trisphenol (PPT) [203] decreased miR-206 expression in MCF-7 cells whereas 2,3-bis(4-hydroxy-phenyl)propionitrile (DPN), an ERβ-selective agonist [204], increased miR-206, pointing to a regulatory loop [162]. Interestingly, miR-206 also reduced β-actin. The authors of this report called miR-206 a “tumor suppressor” and found that miR-206 was higher in ERα-negative MDA-MB-231 cells [162], offering a mechanism, in addition to ERα promoter methylation [205-209], for reducing ERα expression in MDA-MB-231 cells.

A study identifying miRNAs expressed in myometrial and leiomyoma smooth muscle cells (MSMC and LSMC) using microarray and real time PCR reported that E_2_ inhibited the expression of miR-21 in MSMC and LSMC, whereas E_2_ increased and inhibited miR-26a in MSMC and LSMC, respectively [210]. In contrast, ICI 182,780 increased the expression of miR-20a and miR-21 in MSMC and LSMC, and miR-26a in MSMC, while inhibiting the expression of miR-26a in LSMC [210]. No mechanistic studies or mRNA target gene studies were performed to identify the mechanism(s) involved in these cell-specific differences in miRNA regulation by E_2_ and ICI or their downstream targets.

To identify E_2_ regulated miRNAs in a classical estrogen-responsive human breast cancer cell line, we treated ERα-positive MCF-7 cells with 10nM E_2_ or EtOH (vehicle control) for 6 h to identify primary E_2_ target miRNAs. RNA was harvested, labeled either with Cy3 or Cy5, and hybridized with two identical, dual-color miRNA microarrays from LC Sciences. This array contained probes to detect mature miRNA sequences as well as precursor (pre)-miRNAs in the Sanger miRNA registry 7.0 (http://microrna.sanger.ac.uk/ sequences/). The differentially expressed transcripts that were consistent on both chips are summarized in Table **1**. 38 miRNA genes were regulated by E_2_: 9 were reduced and 29 were increased. A summary about what is known about each of these E_2_-responsive miRNAs in terms of breast cancer and estrogen-responsiveness is included in Table **1**.

### miRNAs Regulating ER Expression or Activity

miRNAs can influence estrogen-regulated gene expression by directly reducing ERα mRNA stability or translation. Four miRNAs have been reported to reduce ERα protein levels (Fig. **3**). Two miR-206 recognition sites were identified in the 3’UTR of ERα and transfection of an expression vector for miR-206 in MCF-7 cells reduced both mRNA and protein levels of ERα [162]. Treatment of MCF-7 cells with 1nM E_2_ or the ERα agonist PPT (10nM) reduced miR-206 levels by ~ 80%. In contrast the ERβ agonist DPN (10nM) increased miR-206 expression by ~ 60%. Interestingly, the investigators found that miR-206 levels were significantly higher in ERα-negative MDA-MB-231 cells than in MCF-7 cells, suggesting a mechanism for miR-206 in repressing ERα protein levels in MDA-MB-231 cells. The authors suggested that miR-206 may function in a mutually negative feedback loop to temporally regulate ERα expression and ductal/lobuloalveolar proliferation [162]. More recent studies showed that miR-206 is inversely correlated with ERα expression, but not ERβ, in human breast tumors [211]. Further, transfection of MCF-7 human breast cancer cells with an expression plasmid for pre-miR-206 reduced ERα mRNA expression ~ 25%, reduced the basal expression levels of PR, cyclin D1, and pS2 (all well-established ERα-regulated genes), and inhibited cell proliferation with or without E_2_ [211].

miR-221/222 was recently reported to be higher in ERα negative than ERα positive breast cancer cell lines and human breast tumors [212]. Two miR-221 and miR-222 seed elements were identified in the 3’UTR of ERα and transfection of miR-221 and miR-222 suppressed ERα protein, but not mRNA in ERα positive MCF-7 and T47D cells. Conversely, knockdown of miR-221 and miR-22 in ERα-negative MDA-MB-468 partially restored ERα protein expression and increased tamoxifen-induced apoptosis [212].

miR-22 regulates ERα protein expression in a pancreatic cancer cell line [213]. In a study to identify curcumin gene targets, curcumin increased miR-22 by 65% in BxPC-3 human pancreatic carcinoma cells [213]. One of the predicted 3’UTR gene targets of miR-22 was ESR1 (ERα) [213]. Follow-up studies showed that curcumin reduced ERα protein expression in BxPC-3 cells and that transfection of an antisense RNA oligonucleotide of miRNA-22 into BxPC-3 cells increased ERα protein by ~ 1.9-fold. Thus, miR-22 regulates ERα protein levels and the authors suggest a role for ERα as anti-tumorigenic in pancreatic cancer.

miRNAs can affect estrogen-regulated gene expression by reducing the expression of the coactivator SRC-3/AIB1/ NCOA3. miR-17-5p was demonstrated to inhibit translation of SRC-3/AIB1/NCOA3 [194]. Transfection of CHO-K1 cells with ERα and miR-17-5p inhibited E_2_-stimulated ERE-driven luciferase reporter activity by 50%. This report also demonstrated that transfection of MCF-7 cells (which do not express miR-17-5p) with miR-17-5p reduced E_2_-induced proliferation and E_2_-induced endogenous cyclin D1 transcription [194].

### E_2_ Regulation of Ago2 and ERα in Human Breast Cancer Cell Lines

Argonaut-2 (Ago2), the catalytic subunit of the RISC complex that mediates miRNA-dependent cleavage/degradation in mammals [154, 170, 214], expression is higher in ERα-negative, HER2-positive (basal) than ERα-positive/HER2 negative (luminal) human breast cancer cell lines and tumors [14]. E_2_ and the ERα-agonist PPT, but not the ERβ-agonist DPN, increased Ago2 protein expression in MCF-7 cells [14]. Further studies showed that EGF acts through the MAPK pathway to increase Ago2 protein stability, but there were no studies examining the mechanism by which E_2_ and PPT, presumably through ERα, increase Ago2 protein levels. Surprisingly, Ago2 overexpression in MCF-7 cells increased ERα protein levels by 3-fold, despite also increasing miR-206 that reduces ERα. The authors concluded that this “discordant” finding indicates that there is a greater concentration of miRNAs than target proteins involved in ERα suppression than those that target ERα itself” [14].

## CONCLUSION

**Fig. (1):**
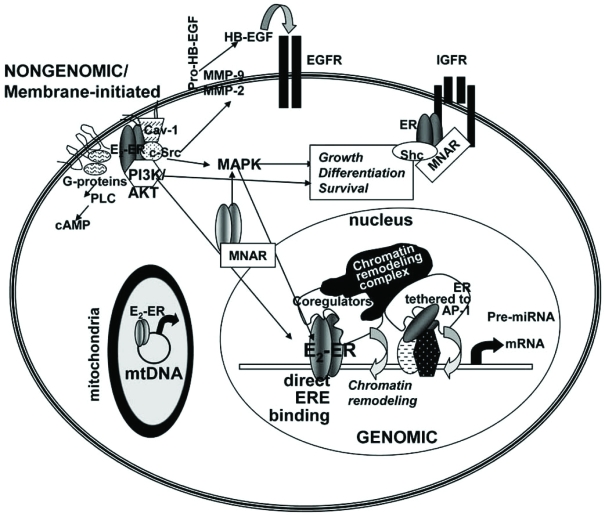
Cell model showing genomic and nongenomic activities of ERα and ERβ. ERα and ERβ are located in the cytoplasm and nucleus, bound to Caveolin-1 in caveolae in the plasma membrane and inside mitochondria. For genomic (nuclear) ER activity, E_2_ binds and activates ER resulting in dimerization, ERE binding or interaction with other transcription factors, *e.g.* AP-1 bound to DNA, coregulator and chromatin remodeling complex recruitment, chromatin remodeling, and increased transcription of target genes. For nongenomic/membrane-initiated estrogen signaling, E_2_ binds ERα in caveolae in the plasma membrane [112, 215]. ERα interacts with G-proteins, the p85 subunit of PI3K, c-Src, and Cav-1 to initiate PI3K/AKT and MAPK signaling cascades [61, 216]. ERα interacts with MNAR [127] and Shc [89] in the cytoplasm. ERα interacts with the EGF- and IGF-1 receptors in plasma membranes. In mitochondria, ERβ interact with the D-loop of mtDNA [217, 218].

**Fig. (3):**
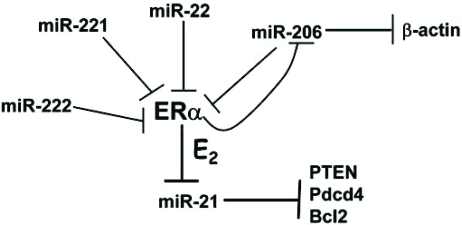
MiRNA regulation of ERα and ERα regulation of miRNA expression. MiRNAs that inhibit ERα protein expression are shown. E_2_-ER represses miR-206 [162] and miR-21 [202, 210] expression. Target genes of miR-21 are PTEN, Pdcd4, and Bcl2 [193]. MiR-206 also represses β-actin expression [162].

Estrogen signaling plays a critical role in regulating reproduction, lactation, bone density, cardiovascular function, neuronal signaling, immune function, and homeostasis in a wide variety of tissues. The reduction in serum E_2_ in postmenopausal women is involved in a number of age-associated disorders. Research on the mechanisms by which E_2_ and other estrogens regulate diverse physiological effects has established both genomic and nongenomic mechanisms involving ERα, ERβ, and GPR30 in signal transduction (Fig. **1**). miRNAs are small, non-coding RNAs that bind to the 3’ UTR of target mRNAs and either block the translation of the message or bind the ORF and target the mRNA transcript to be degraded. Although there are a number of studies identifying miRNA changes in breast tumors and comparing ERα-positive *versus* ERα-negative miRNA signatures for their potential use as biomarkers, there are few studies identifying E_2_-responsive miRNAs in any normal or neoplastic tissues or cell models. In those few studies that have identified E_2_-induced alterations in miRNA expression, there is little, if any, mechanistic detail elaborated for the E_2_ effect (s) on miRNA expression. Further, it appears that E_2_ regulates miRNA expression in a cell-type-dependent manner. Thus, identification of E_2_-regulated miRNAs and the function of miRNAs within specific tissues and cells still remains to be determined.

## Figures and Tables

**Fig. (2):**
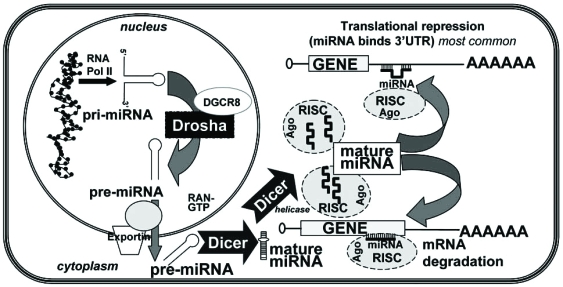
Model of miRNA biogenesis and function. Primary transcripts of microRNAs (pri-miRNAs) are transcribed by RNA polymerase II, processed by the RNAse III enzyme, Drosha and its cofactor DGCR8, to precursor microRNAs (pre-miRNAs) and are then exported from the nucleus by Exportin/RAN-GTP [150]. In the cytoplasm, pre-miRNAs are processed by the RNAse III enzyme, Dicer to form mature ~22 nt transiently double-stranded miRNA duplexes that are transferred to Argonaute proteins (Ago1, Ago2, Ago3, and Ago4 [152]) in the RNA-induced silencing complex (RISC), leading to unwinding of the duplexes to form single stranded miRNAs. The mature miRNAs bind either to the 3’ untranslated region (3’ UTR) or to the open reading frame (ORF) of its target mRNA [153-156]. Binding of miRNA/RISC complex with the 3’UTR causes translational repression [16]. Thus, miRNA-mRNA 3’UTR interaction results in a decrease in target protein, not mRNA.

**Table 1. Comparison of miRNA Expression in MCF-7 Cells Treated for 6 h with E2Versus EtOH:** MCF-7 cells were grown in dextran-coated charcoal-stripped, phenol red free IMEM medium for 48 h prior to a 6 h treatment with ethanol (EtOH, vehicle control) or 10 nM E_2_. RNA was isolated using miRVana and sent to LC Sciences for miRNA microarray analysis. All miRNA gene changes included in this table are statistically significant as analyzed by LC Sciences. Negative values indicate decreased expression and positive values indicate increased expression with E_2_. NS = no significant change in miR gene expression with E_2_-treatment.

miRNA	Log 2 (E_2_/EtOH)	Comments Regarding the Possible Connection of the Identified miRNA Gene with Breast Cancer and/or Estrogenic Responses. *Bone Fide* Targets of miRNAs are Indicated
let-7a	-0.3	Expressed in ERα positive human breast tumors [22]. Expression was higher in ERα+ than ERα- tumors [12]. Expressed in ZR-75, MCF-7, BT474, SK-BR-3, and MDA-MB-231 breast cancer cells [219]. Expression of Let-7 family members is reduced in ‘breast cancer stem cells’(CD44+CD24−/low) [220]. Targets of let 7 are Ras [170] and Caspase-3 [221].
let-7cc	0.3	Expression was higher in PR+ *versus* PR- human breast tumors [22] and in ERα+ than ERα- tumors [12]. let-7c increased TRAIL-induced caspase 3 activation in MDA-MB-453 breast cancer cells and let-7c was predicted to target CD95L [222].
let-7dd	0.25	let-7d was increased in acute promyelocytic leukemia patients[223].
let-7f	-0.25	let-7f was > in node negative *versus* positive human breast tumors [22] and higher in ERα+ tumors [12]. let-7f in mammary gland was reduced by E_2_ treatment of female ACI rats [198].
let-7g	1.66	let-7g was expressed in ErbB2 positive human breast tumors [22].
let-7i	1.34	
miR-106b	1.14	Higher in PR+ than PR– tumors, but higher in ERα- than ERα+ breast tumors [12]. miR-106b is overexpressed in breast tumors compared to normal breast and miR-106b reduced p21 mRNA and protein and thus stimulates G1-S cell cycle progression in human mammary epithelial cells [224]. miR-106b in mammary gland was increased by 6 wks of E_2_ treatment of female ACI rats [198].
miR-149	-3.17	miR-149 in mammary gland was increased by 6 wks of E_2_ treatment of female ACI rats [198].
miR-15a	2.32	Higher in low *versus* high tumor stage in human breast tumors [20] and greater in ERα+ than ERα- tumors [22]. Higher in PR+ than PR- tumors [22]. miR-15a is a tumor suppressor [225]. miR-15a negatively regulates Bcl2 at a posttranscriptional level [226].
miR-15b	1.13	
miR-151	0.27	Higher in ERα+/lymph node negative breast tumors from patients with a short time to distant metastasis (TDM) versus those with a long TDM [15].
miR-16	0.79	Higher in ERα+ than ERα- tumors [22]. miR-16-1 negatively regulates Bcl2 at a posttranscriptional level [226]. The miR-16 family negatively regulates cell cycle progression by inducing G0/G1-cell accumulation [227] by reducing CCND1 (cyclin D1), CCND3, CCNE1, and CDK6 [228].
miR-182	0.83	Higher in ERα+ than ERα- human breast tumors and not significantly higher in ErbB2 –*vs*. positive tumors [22] significantly higher in PR+ *vs*. PR– tumors [22]. miR-182 inhibited TRAIL-induced caspase 3 activation in MDA-MB-453 breast cancer cells and miR-182 was predicted to target caspase 3 and FADD [222].
miR-183	0.98	
miR-195	2	Higher in ErbB2- vs. ErbB2 positive tumors [22], but not significantly > in ERα+ than ERα- or PR+ *vs*. PR– tumors [22]. miR-195 expression was increased by hypoxia in MCF-7 cells [229]. miR-195 inhibits CCND1,CCND3, CCNE1, and CDK6 protein expression [228].
miR-200a	2.58	Correlated with ERα status in human breast tumors [22] and was significantly > in ERα+ than ERα- and PR+ than PRbreast tumors [22]. miR-200a is expressed in MCF-7 and other epithelial breast cancer cell lines [230]. miR-200 expression was reduced in tamoxifen-resistant MCF-7 cells [17].
miR-200b	0.7	miR-200b is expressed in MCF-7 and other epithelial breast cancer cell lines [230].
miR-200c	-0.42	miR-200c is expressed in MCF-7 cells [166] and is higher than miR-200a or -200b in MCF-7 [230].
miR-203	1.84	Expression is increased in ovarian, breast and melanoma cancers [178]. Higher xpression in high *versus* low tumor stage in human breast tumors [20] and increased in tamoxifen-resistant MCF-7 cells [17].
miR-20a	0.83	Increased in lung, breast, stomach, prostate, colon, and pancreatic tumors [177]. miR-20a expression was low in MCF-7 and other breast cancer cell lines and overexpression of the miR-17/20 locus in MCF-7 inhibited cell proliferation and cyclin D1 expression [19]. miR-20a was increased in mammary gland after 6 or 12 wks of E_2_ treatment of female ACI rats [198]. Both E_2_ and ICI decreased miR-20a in human endometrial stromal cells [231]. Targets of miR-20a are PCAF, RUNX1, and TGFBR2 [232].
miR-21	-0.14	Significantly up-regulated in tissues or cell lines of breast cancer [20]. overexpressed in all solid tumors (lung, breast, stomach, prostate, colon, and pancreatic) [177]. significantly higher in ERα+ than ERα-, in ErbB2 - *vs*. ErbB2 +, and in PR+ *vs*. PR– breast tumors [22]. Expression was higher in breast tumor compared to adjacent normal breast tissue [233]. miR-21 expression was increased by hypoxia in MCF-7 [229]. miR-21 in mammary gland was increased after 18 wks of E_2_ treatment of female ACI rats [198]. Both E_2_ and ICI decreased miR-21 in human endometrial stromal and glandular epithelial cells, but when combined, miR-21 expression returned to basal [231]. E_2_ suppressed and ICI increased miR-21 in human human myometrial smooth muscle cells [210]. E_2_ inhibited the ICI-induced increase in miR-21 in these cells [210]. E_2_ (75% decrease) and Progesterone (41% decrease) reduced miR-21 expression on the uterus of ovex mice [202]. miR-21 expression was significantly reduced in tamoxifen-resistant MCF-7 cells [17].
miR-23a	0.31	Increased by hypoxia in MCF-7 cells [229]; reduced in tamoxifen-resistant MCF-7 cells [17].
miR-23b	0.32	Increased by hypoxia in MCF-7 cells [229].
miR-25	1.6	Higher in ERα+ than ERα- breast tumors [12] and PR+ *vs*. PR– breast tumors [22], but ot significantly higher in ErbB2-negative *vs*. positive breast tumors [22]. increased in ovarian, breast and melanoma cancers [178]. miR-25 was increased in mammary gland after 6 wks of E_2_ treatment of female ACI rats [198].
miR-26a	0.87	Significantly > in ERα+ than ERα- breast tumors [20, 22]. significantly higher in ErbB2 – *vs*. ErbB2-positive breast tumors [22] and PR+ than PR- tumors [20]. miR-26a expression was increased by hypoxia in MCF-7 cells [229]. Both E_2_ and ICI increased miR-26a in human endometrial glandular epithelial cells, but when combined, miR-26a expression was suppressed below basal [231]. E_2_ and ICI increased miR-26a in human myometrial smooth muscle cells, but each inhibited miR-26a in human leiosarcoma cells [210].
miR-26b	2.07	Higher in ERα+ than ERα- breast tumors [22], but mot significantly higher PR+ *vs*. PR–tumors [20]. Higher in ErbB2 –ve *vs*. positive breast tumors [22]. miR-26b expression was increased by hypoxia in MCF-7 cells [229].
miR-27a	1.73	Higher in ErbB2- vs ErbB2+ and PR+ *vs*. PR- breast tumors [22], but not significantly higher in ER+ *vs*. ER– tumors [20]. miR-27a expression was increased by hypoxia in MCF-7 cells [229].
miR-27b	1.94	Higher in ERα+ than ERα- breast tumors [22], in ErbB2-*vs* ErbB2+ tumors [22], and in PR+ *vs*. PR– tumors [22].
miR-30b	2.17	Higher in node negative *versus* positive human breast tumors [20], higher in ERα+ than ERα-, in ErbB2 - *vs* ErbB+ and in PR+ *vs*. PR– breast tumors [22]. miR-30b expression was increased by hypoxia in MCF-7 cells [229].
miR-320	-0.84	
miR-328	-3.92	Overexpression of miR-328 in A431 human epithelial carcinoma cells reduced cell adhesion, aggregation, and migration by repressing CD44 expression [234].
miR-342	-0.26	Higher in ERα+ than ERα- ,in ErbB2 – *vs*. ErbB2+ , and in PR+ *vs*. PR– tumors [22]. miR-342 expression was reduced in tamoxifen-resistant MCF-7 cells [17].
miR-365	1.47	Decreased by E_2_ treatment in female ACI rat mammary gland [198].
miR-423	-1.49	
miR-489	0.59	Metastasis suppressor: decreased expression in metastatic sublines of MDA-MB-231[18]. miR-489 expression was reduced in tamoxifen-resistant MCF-7 cells [17].
miR-7	1.84	
miR-92	0.45	Higher in ERα+ than ERα- and in PR+ *vs*. PR– breast tumors, but not higher in ErbB2- *vs*. ErbB2+ tumors [22]. miR-92 was increased in mammary gland after 6 wks of E_2_ treatment of female ACI rats [198]. miR-92 is in the miR-17/20 cluster and overexpression of the miR-17/20 cluster in MCF-7 cells inhibited basal and E_2_-stimulated cell proliferation and cyclin D1 transcription [19].
miR-98	1.55	Overexpressed in breast tumor compared to adjacent normal breast tissue [233].
